# Behavioral Responses of the Common Bed Bug to Essential Oil Constituents

**DOI:** 10.3390/insects12020184

**Published:** 2021-02-21

**Authors:** María A. González-Morales, Martín Terán, Alvaro Romero

**Affiliations:** 1 Department of Entomology and Plant Pathology, North Carolina State University, Campus Box 7613, Raleigh, NC 27695, USA; magonz23@ncsu.edu; 2 Critter Control, Marietta, GA 30062, USA; martinteran67@gmail.com; 3 Department of Entomology, Plant Pathology and Weed Science, New Mexico State University, Las Cruces, NM 88003, USA

**Keywords:** *Cimex lectularius*, resurgent urban pest, avoidance behavior, feeding assay, choice test, video-tracking

## Abstract

**Simple Summary:**

Bed bugs (*Cimex lectularius* L.) are blood-sucking insects that have emerged worldwide in the last two decades causing serious public health and economic impact. Today, control of bed bug infestations relies on the use of synthetic insecticides, but their frequent use has led to the development of resistance in bed bug populations. Therefore, there is a growing demand for the development of safer, green, and more effective tools for bed bug control. Plant-derived pesticides are part of the proposed “green” methods for bed bug control. We evaluated behavioral responses of bed bugs to essential oil constituents (EOCs) and detected that bed bugs did not rest on areas treated with geraniol, eugenol, citronellic acid, and carvacrol. Barriers of these constituents did not deter bed bugs from reaching warmed blood meal and feeding. Our results show that novel formulations of natural product insecticides that include geraniol, eugenol, carvacrol, or citronellic acid have potential to repel bed bugs. However, little benefit of protection against bed bug bites can be expected when EOC-based products are applied to items present in close proximity to a sleeping host such as mattress covers, liners, or around the bed.

**Abstract:**

Botanical-derived pesticides have arisen as an attractive alternative to synthetic insecticides to effectively manage infestations of bed bugs (*Cimex lectularius* L.). While information on contact, residual, and fumigant toxicity of plant-essential oils against bed bugs have been recently published, there is a gap of information regarding the repellent activity of these products and their constituents. Identification of essential oil constituents (EOCs) with repellent activity will help develop potentially efficacious essential oil-based formulations for use in bed bug management programs. In this study, we first screened fresh and 24 h-aged residues of geraniol, eugenol, carvacrol, thymol, citronellic acid, linalool, menthone, trans-cinnamaldehyde, α-pinene, β-pinene, and limonene for avoidance behavior from individual bed bugs with a video-tracking system. Six EOCs, geraniol, eugenol, citronellic acid, thymol, carvacrol, and linalool were further evaluated overnight in choice tests to determine whether 24-h aged residues were still avoided by groups of bed bugs. While bed bugs avoided resting on filter papers treated with 24-h aged residues of geraniol, eugenol, citronellic acid, and carvacrol, bed bugs aggregated in areas treated with linalool-aged residues. Barriers of EOCs did not prevent bed bugs from reaching a warmed blood source and acquiring blood meals. Our results show that novel formulations of natural product insecticides that include geraniol, eugenol, carvacrol, or citronellic acid have potential to repel bed bugs. The presence of host-associated cues might interfere with these responses.

## 1. Introduction

Bed bugs (*Cimex lectularius* L.) are blood-sucking ectoparasites that usually feed at night [1]. During daytime, bed bugs remain hidden in cracks and crevices around sleeping and resting areas of its host. Bed bugs become highly active after the onset of darkness in search for a host [2] which they locate following visual, olfactory, and thermal signals that are detected by the sensory system located primarily on the insect’s antenna [3]. It is during this time that insects might encounter semiochemicals and chemical insecticides.

In the last two decades, bed bugs have resurged worldwide becoming one of the most challenging urban pests to control [4]. The importance of bed bugs to public health in large part is due to skin reactions caused by their bites [5] and the anxiety, stress, sleeplessness, and social distress in people who have been continuously exposed to bed bug infestations [6]. Treatment with synthetic insecticides, such as pyrethroids and neonicotinoids, is the primary tool for bed bug control today but their intense use has led to the emergence of populations highly resistant to these chemical groups in some parts of the world [7,8,9]. The difficulty in eliminating resistant bed bug populations could further increase the escalation of infestations worldwide.

Integrated pest management (IPM) strategies have been proposed for long-term management of bed bug infestations [10]. This approach includes the combined use of chemical methods (liquid sprays, dusts, and fumigants), and non-chemical tools (vacuuming, heat, steam, washing and drying, low temperatures, exclusion with mattress encasements). Challenges in reducing bed bug incidence and densities, particularly in multiunit housing communities, have prompted a community-wide bed bug management program consideration. This program includes proactive detection of bed bugs and training for staff members and residents regarding biology, behavior and prevention of infestations [11,12]. While many prevention methods have been proposed in these programs (e.g., monitors, mattress encasements, use of desiccant dusts, structural modification through sealing of cracks of crevices) [13], these tools are meant to prevent infestations from becoming established, and not for personal protection or reduce the passive transportation of bed bugs from one place to another. The application of repellents could be an effective method to prevent bed bug bites, avoid hitchhikers while visiting infested places, or reduce the spread of infestations through personal items or furniture [14]. Information on the efficacy and use of repellents against bed bugs is limited. N,N-Diethyl-3-methylbenzamide (DEET), the most widely used insect repellent, has been evaluated using in vivo [15] and in vitro systems [14,16,17] providing greater efficacy and extended residual activity. However, there is a growing concern about the public’s negative perception about the impact of DEET on human health [18]. Alternative compounds that have demonstrated repellent activity against bed bugs include 3-methyl-5-hexyl-2-cyclohexenone, propyl dihydrojasmonate, and γ-methyl tridecalactone [14].

Natural insecticides, especially plant essential oils, have been used extensively to interrupt host seeking of blood-feeding insects [17,19,20]. These compounds are attractive alternative tools to synthetic chemical repellents because they are considered to have low toxicity to humans, animals and the environment [18]. Because of their minimum risk, the U.S. Environmental Protection Agency (EPA) have exempted some plant-derived compounds from full registration [Section 25(b) of the Federal Insecticide, Fungicide, and Rodenticide Act of United States] [21]. Reduced registration requirements by EPA has led to a flood of essential-oil based products available commercially for indoor use. Many of these compounds have been evaluated in the laboratory for repellent activity against urban pests [22,23,24]. Essential oil constituents such as geraniol and citronellol had repellent activity on *Triatoma rubida* (Uhler)*, T. protacta* (Uhler)*,* and *T. recurva* (Stal) (Hemiptera: Reduviidae) [25], while carvacrol, eugenol, and geraniol repelled *Rhodnius prolixus* (Stål) and *T. infestans* (Klug) (Hemiptera: Reduviidae) [26]. Thymol was reported as having repellent activity against *Blatella lateralis* (Walker) (Blattodea: Blattidae) [24]. In bed bugs, several studies have evaluated the contact toxicity [27,28,29,30,31], fumigant toxicity [29,32,33], and residual toxicity [34,35] of plant-derived pesticides, but few have studied in detailed their repellency. Anderson et al. [36] demonstrated the efficacy of three naturally occurring repellents, para-menthane-3,8-diol, delta dodecalactone, and gamma dodecalactone to prevent bed bugs from hiding in substrate-treated areas. Catnip oils also exhibited high repellency activity against bed bugs, but their residual effect was shorter than DEET [37]. Sharififard et al. [38] conducted laboratory evaluations with essential oils of oregano *Origanum vulgare* and demonstrated 100% repellency against bed bugs up to 24 h. In choice tests, bed bugs seemed to avoid contacting areas treated with lethal doses of two essential oil-based products, EcoRaider^®^ and Bed Bug Patrol^®^ [27]. The latter three studies did not characterize the repellency or avoidance induced by major constituents of the essential oils in bed bugs. There is also an anecdotal report of the lack of repellency activity of cedar and peppermint oil against bed bugs [14], which might be explained by the absence of essential oil constituents (EOCs) with repellent properties in the composition of these essential oils. Identification of EOCs with repellent activity will help develop potentially efficacious essential oil-based formulations. Recently, medium-chain length fatty acids from coconut oil exhibited strong and lasting repellent activity against various blood-sucking arthropods, including bed bugs [17]. The easing of the US government requirements for plant product registration, the relative low production cost of many of these materials, and the perception by consumers as “green”, make these products likely to be adopted and incorporated into IPM programs for bed bugs.

The objective of our study was to characterize behavioral responses of bed bugs to (EOCs) that are common active ingredients of essential oil products using a video-tracking system and supplemented with a laboratory choice test. We also assessed whether bed bugs would crawl over areas treated with (EOCs) to reach a heat source and take a blood meal. Our results show that essential oil formulations with geraniol, eugenol, carvacrol and citronellic acid have the potential to protect personal belongings from bed bugs, but the presence of heat, and other host associated-cues, might interfere with these responses.

## 2. Materials and Methods

### 2.1. Insects

Bed bugs were obtained from a colony maintained at 25 °C, 70 ± 5% relative humidity, and a photoperiod of 12:12 h (light:dark) [2]. This colony was originally established from bed bugs collected in an apartment in Jersey City, NJ, USA in 2008. Insects were fed in the laboratory through a parafilm membrane feeder with defibrinated rabbit blood heated to 37 °C by a circulating water bath [39]. Experimental insects were tested unfed, 7 days after adult emergence.

### 2.2. Chemicals

High purity (>97%) of DEET and the EOCs, geraniol, eugenol, carvacrol, thymol, citronellic acid, linalool, menthone, trans-cinnamaldehyde, α-pinene, β-pinene, and limonene were obtained from Sigma-Aldrich (St. Louis, MO, USA). The chemicals were diluted to 1% in acetone (99.7% purity; Fisher Scientific, Fair Lawn, NJ, USA).

### 2.3. Tracking of Individual Bed Bugs

#### 2.3.1. Arenas

**Figure 1:**
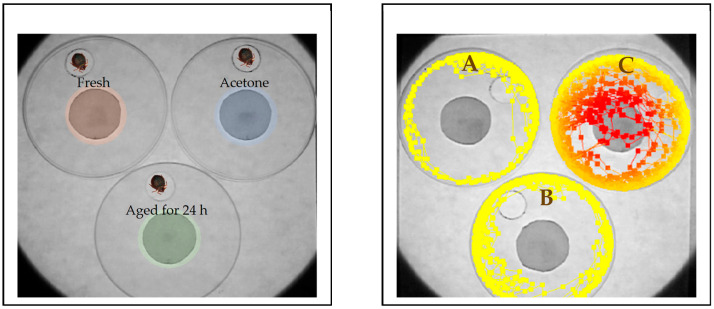
Left, arrangement of experimental arenas for simultaneous tracking of responses from individual bed bugs to essential oil constituents. Right, an example of tracks of bed bugs avoiding fresh residues (**A**), or residues aged for 24 h (**B**). (**C**) is tracks of a bed bug interacting with a control arena (acetone-treated area); red tracks represent approximation of the bed bug to the acetone-treated area, indicating that the insect did not exhibit avoidance behavior.

Tracking of individual bed bugs was conducted using the bottoms of plastic petri dishes (9 cm) with a small hole (1 cm) cut into the plastic (Figure 1). These dishes were flipped on a square glass (23 cm × 23 cm) whose floor was covered with white filter paper (Whatman no. 2) (Figure 1). Each petri dish enclosed a 3-cm diameter filter paper placed in the center and impregnated with 100 µL of each solution at 1%, or acetone only. These circles were previously allowed to dry for 1 h (fresh residues) or aged for 24 h in a fume hood. Each tracking included arenas treated with acetone, the fresh compound, or aged for 24 h (Figure 1). Insects were acclimated to the environment for 15 min by restricting them in a shell vial (21 mm diameter × 70 mm height) which was placed inverted through the opening in the petri dish bottoms (Figure 1, left). Insects were released by lifting up the shell vial. Recordings were conducted under ambient temperature (25 ± 2 °C) and relative humidity (40 ± 10%) for 10 min within the first three hours of the scotophase, a time in which bed bugs display enhanced locomotor activity [2].

#### 2.3.2. Video Tracking System

A near-infrared (NIR) camera (series acA1300-60 gm NIR camera, Basler^®^ ace; Exton, PA, USA) outfitted with a lens (C-mount 4–8 mm varifocal megapixel CCTV lens, model# H2Z0414C-MP, Computar^®^; Torrance, CA, USA) and (infrared) IR filter (Infrared 850 light filter, Heliopan^®^, North White Plains, NY, USA) was used to record bed bug activity in the arenas under dark conditions. The camera was positioned approximately 58 cm directly above the center arena. Light for the recordings was provided by two IR illuminators (AT-8SB 850 mm, 130_, AXTON^®^, North Salt Lake, UT, USA). EthoVision XT version 11.5 software (Noldus Information Technology Inc. Leesburg, VA, USA, [40] was used to capture video images and to track the bed bugs during 10-min bioassays. EthoVision XT virtually facilitates the marking of each treated circle. The software transformed bed bug tracking activity into several variables, but only two variables were used to characterize behavioral responses: “distance” (cm) to treated area” and “number of visits” to the treated area. The same variables were calculated from the activity of bed bugs recorded in control arenas (acetone treated). Comparisons of “distance” were made with one-way analysis of variance (ANOVA) followed by Dunnett’s test for mean separation within treatments (control vs. fresh residues, or control 24-h aged residues), or Tukey’s pairwise test for mean separation among treatments. Data from “number of visits” failed the normality test (Kolmogorov–Smirnov test, <0.05) and comparisons within and among treatments were made with the nonparametric Mann–Whitney test and a Bonferroni-adjusted α level of 0.0167. [41].

### 2.4. Choice Tests with 24-h Aged Residues

Six EOCs, geraniol, eugenol, citronellic acid, thymol, carvacrol, and linalool were further evaluated with a choice test to assess avoidance responses from bed bugs during a 14 h-period. The selection of these EOCs was based on the lower visitation rates by bed bugs to 24-h aged residues detected in the tracking experiment (Table 1). Insects were offered two tents made of filter paper (15 × 12 mm, Whatman no. 2) folded in the middle to offer a tent-like shelter of 15 mm length by 5 mm height with two open ends. Group responses were carried out in flat-bottomed Pyrex bowls (12.4 cm diameter by 6.0 cm height; Corning, Corning, NY, USA) whose surfaces were covered with a white filter paper (110 mm diameter; Whatman no. 2), fixed to the glass with double-sided tape. After each assay, papers were removed and bowls were rinsed with acetone. During the photophase, arenas were illuminated with 40-W fluorescent tubes placed 60 cm above the arena surfaces, which provide a light intensity of ~660 lux. Under these conditions, bed bugs would seek harborages during the day. Groups of ten bed bugs (1:1 sex ratio) were offered tents that had been treated with 50 µL of 1% solution of geraniol, eugenol, citronellic acid, thymol, carvacrol, or linalool. 1% DEET was used as a positive control while acetone as negative control. Treated tents were aged 24 h before use. Six replicates were performed for each compound. All assays lasted 14 h (from 18:00 to 08:00 h the next day) with the following light–dark regimen: lights off at 18:00 h and lights on at 06:00 h (the same light cycle used during rearing). Room temperature remained at 24 ± 2 °C. Insects were acclimated to the environment for 15 min by restricting them in a shell vial (21 mm diameter × 70 mm height) which was placed inverted in the center of the arena. Insects were released by lifting up the shell vial within the first hour of the scotophase. At the end of the test, the location of insects resting on a tent or wandering in the arena was recorded. The number of responses was analyzed by a binomial test with exact two-tailed *p* values, with the null hypothesis that the tent were chosen with equal probability.

### 2.5. Feeding Test

**Figure 2:**
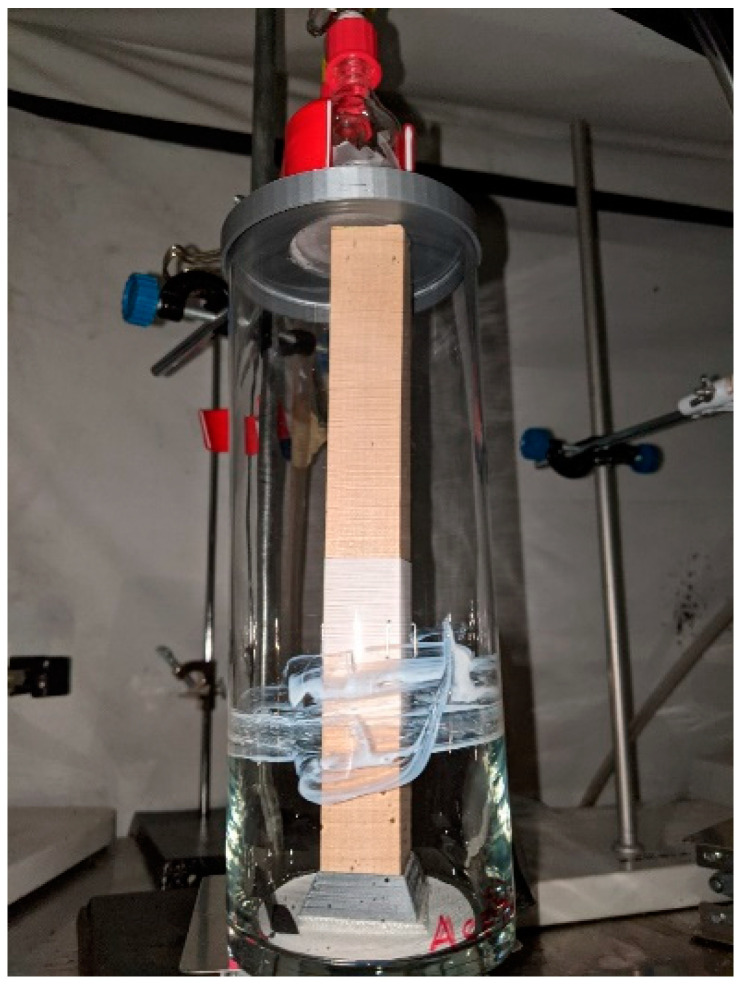
Feeding assay to evaluate responses of bed bugs to essential oil constituent barriers. Bugs needed to cross the treated barrier to take a blood meal.

This experiment was conducted in a glass cylinder (height: 22 cm, diameter: 8 cm, Hobby Lobby, Oklahoma City, OK, USA) (Figure 2); the open end of each container was covered with a screened lid (500 µm plankton netting, Bioquip Products, Rancho Dominguez, CA, USA). This fabric top made contact with a parafilm-membrane feeder previously described. Insects reached the heated surface by crawling up a square wood post (2.5 cm each side, 21 cm height). A paper strip (10 cm wide by 5 cm height, Whatman no. 2) was impregnated with an EOC-acetone solution (600 µL of 1%, geraniol, eugenol, citronellic acid, carvacrol, or acetone), wrapped around the stick, 5 cm away from the base, and attached with staples. To reach the blood source (37 °C), the bed bugs needed to cross the EOC-treated band. Twenty insects (1:1 sex ratio, females and males were morphologically differentiated according to Usinger [1]; 3 replicates) were restricted in a shell vial (21 mm diameter × 70 mm height) and acclimatized for one hour in the bottom of the cylinder, before turning on the water circulator system that warmed the blood held in the glass feeder. The bugs were released under the similar conditions used in choice tests. The number of fed and unfed insects after 12 h was recorded for each treatment and analyzed with a chi-square goodness-of-fit-test [41].

## 3. Results

### 3.1. Responses of Insects to Essential Oil Constituent Impregnated Areas

Bed bugs in arenas with fresh or 24-h aged residues of geraniol (F = 24.86, df = 2, *p* < 0.001), or eugenol (F = 48.68, df = 2, *p* < 0.0001) were significantly more distanced from EOC-treated areas than bed bugs in control arenas (Table 1). Similarly, bed bugs significantly avoided visiting areas impregnated with fresh or 24-h aged residues of geraniol (fresh residues: *U* = 154.5, *p* = 0.0002; aged residues: *U* = 147.5, *p* = 0.0015), eugenol (fresh residues: *U* = 155, *p* = 0.0002; aged residues: *U* = 150.5, *p* = 0.0015), thymol (fresh residues: *U* = 145.0, *p* = 0.0131; aged residues: *U* = 147.5, *p* = 0.0141, and citronellic acid (fresh residues: *U* = 146.0, *p* = 0.003; aged residues: *U* = 150.5, *p* = 0.0014), when compared with control arenas (Table 1). Only fresh residues of carvacrol (*T* = 4.184, *p* = 0.0011), thymol (*T* = 4.836, *p* = 0.0013), and trans-Cinnamaldehyde (*T* = 3.708, *p* = 0.0075) kept bed bugs significantly more distanced than bed bugs in control arenas (Table 1). While fresh residues of linalool were significantly less visited by bed bugs (*U* = 144.0, *p* = 0.0163), aged residues of α-pinene had the highest rate of visitation among all components tested (*U* = 135.0, *p* = 0.0445). Analysis of distance among treatments showed that bed bugs were more distanced from areas treated with thymol than other EOCs with both residues, fresh (F_10,73_ = 8.19; *p* < 0.0001) and 24-h aged (F_10,73_ = 3.31; *p* < 0.05). Number of visits of bed bugs to areas treated with fresh residues were significantly different among EOCs (H (10) = 31.93, *p* < 0.001)), with significant lower visitation rates to fresh residues of eugenol and citronellic acid (Table 1). Similarly, 24-h aged residues of geraniol, eugenol, thymol, and citronellic had significantly lower visitation rates than other EOCs ((H (10) = 39.80, *p* < 0.001)).

### 3.2. Choice Tests

**Figure 3:**
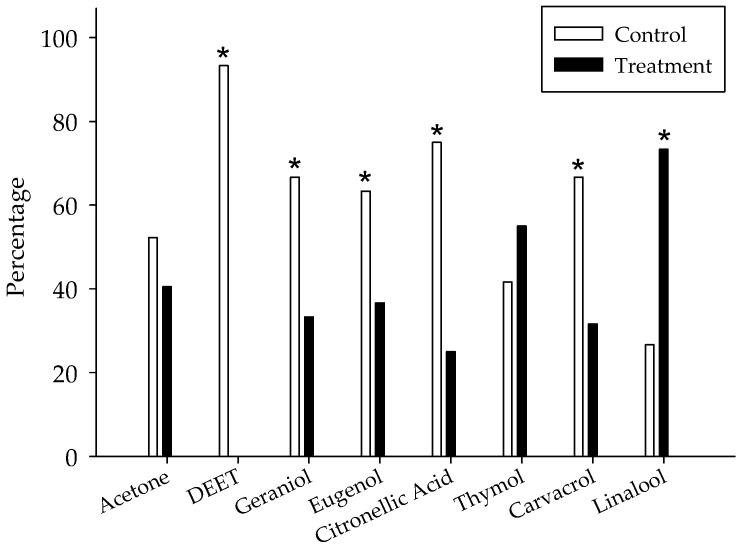
Bed bug preference (five females, five males) for a paper tent impregnated with essential oil constituents (1%) aged for 24 h vs. a tent impregnated with acetone. After a 14-h test period, the number of insects resting on any of the treatment harborages or wandering on the arena was recorded. * Significant differences between control and EOC-treated tents (*p* < 0.05; *N* = 6).

There was no preference of bugs for tents treated with acetone or untreated (Figure 3). The bugs significantly preferred to settle in acetone-treated tents rather than in tents with 24-h aged residues of DEET (*p* < 0.0001), geraniol (*p* = 0.0135), eugenol (*p* = 0.047), citronellic acid (*p* = 0.0001); and carvacrol (*p* = 0.0086) (Figure 3). While bugs did not significantly avoid thymol-treated tents (*p* = 0.3581), groups of bed bugs assembled significantly more in linalool-treated tents (*p* = 0.0004) (Figure 3).

### 3.3. Responses of Host-Seeking Insects to EOCs Barriers

There were significant differences in the percentage of fed insects between control and citronellic acid (65 versus 36.7%, χ^2^ = 4.73, *p* = 0.03). No significant differences were found between control and geraniol (80 versus 58.3%, χ^2^ = 2.03, *p* = 0.154), control and eugenol (80 versus 65%, χ^2^ = 0.93, *p* = 0.335), and between control and carvacrol (65 versus 48.3%, χ^2^ = 1.47, *p* = 0.225).

## 4. Discussion

While essential oils and EOCs have demonstrated excellent contact and fumigant toxicity against bed bugs [29,32,35], there is still limited information on the repellency of these compounds when applied to personal belongings or directly to skin to protect a host from bites. However, skin-applied repellent products can be sticky and therefore not suitable for use before going to bed. In addition, there are health concerns due to the prolonged and repeated exposure of the host to these compounds [42]. The most practical option to deter bed bugs from biting a host could be to treat fabric or areas in the proximity of the bed with repellents. Similarly, passive transportation of bed bugs in furniture and personal belongings might also be prevented by treating these items with effective and long-lasting repellents. Therefore, characterization of behavioral responses of bed bugs to plant-derived compounds could lead to the identification of compounds that help reduce bed bug biting activity and the spread of bed bugs.

We initially characterized the locomotor responses of individual bed bugs to EOCs using the tracking system Noldus EthoVision XT. The system tracked simultaneously and separately, the activity of individual bed bugs interacting with acetone, fresh or 24-h aged residues of EOCs during 10 min (Figure 1). The variables that yielded the most informative and consistent results about repellency toward EOCs were distance and number of visits to treated areas. Number of visits in Table 1 showed that bed bugs made contact with areas treated with fresh residues of all of the tested EOCs. However, bugs interacting with fresh residues of geraniol, eugenol, carvacrol, thymol, and trans-cinnamaldehyde tended to avoid both getting close to and visiting treated areas, while bugs in arenas with fresh residues of citronellic acid and linalool had a reduced visitation rate to treated areas. These avoidance responses could have been the result of bed bugs moving away from the treated area either because of irritancy (after contacting the treated area) or because repellency (after perceiving the EOCs at some distance). Plant EOCs have shown to cause contact toxicity against insect pests including cockroaches, kissing bugs, and bed bugs [24,26,29]. In a previous study on bed bugs, carvacrol, thymol, citronellic acid, eugenol, and geraniol had the highest contact toxicity, with some of them inducing increased locomotor activity [29]. From the 11 EOCs tested in our study, seven components elicited avoidance responses from bed bugs, five of which were terpenoids (geraniol, carvacrol, thymol, citronellic acid, linalool), one was a phenylpropane (eugenol), and one was an aldehyde (trans-Cinnamaldehyde). Plant essential oils that contain high concentrations of effective constituents identified in this study are java citronella oil (geraniol, citronellic acid), clove oil (eugenol), and red thyme (carvacrol, thymol, linalool) [24], and cinnamon oil (trans-Cinnamaldehyde) [43]. While several studies have shown repellent activity of essential oils against other urban pests [22,24,25], there is only a few of repellency reports in bed bugs [17,27,28,38]. Essential oils of oregano *Origanum vulgare,* containing carvacrol and thymol, repelled bed bugs up to 24 h [38]. Of nine essential oil-based products that are commercially-available for bed bug control [27], only EcoRaider^®^ (active ingredients: geraniol (1%), cedar extract (1%), and sodium lauryl sulfate (2%) and Bed Bug Patrol^®^ (active ingredients: clove oil (0.003%), peppermint oil (1%), and sodium lauryl sulfate (1.3%) were reported to be effective as repellents against bed bugs [27]. It is very likely that essential oils containing effective EOCs have reduced repellency properties, or not have it at all, due to the presence of other EOCs that affect their bioactivity.

The mechanisms by which EOCs cause toxicity in insects include interaction with various neurotransmitter receptors at the nervous system level, and the extent of the toxicity vary according to the structural and chemical properties of the components [44]. The tendency of terpenoids to cause more toxicity and behavioral effects than other non-terpenoid constituents have been reported in other bed bug’s studies [29] and suggest that terpenoids are important in the development of new repellents for use in bed bug control.

Recent electrophysiological studies on bed bug’s thoracic ganglions have shown that, while linalool triggered neuroexcitatory responses, carvacrol, thymol, eugenol, and citronellic acid had neuroinhibitory effects [29]. Geraniol has also been reported to have a similar depressive effect in the abdominal nerve cord of the cockroaches *Periplaneta americana* L. (Blattodea: Blattidae), and *Blaberus discoidalis* (Serville) (Blattodea: Blaberidae) [45]. Therefore, all these neurotoxic effects could have contributed to the avoidance behavior when bed bugs contacted the EOC residues. Avoidance behavior to EOCs might also be due to sensorial detection of their volatile constituents by bed bugs, and electrophysiological studies support this. Single cell recordings have showed that botanical-derived repellents trigger action potentials from the D and E1 sensilla olfactory neurons located in antennal sensilla of bed bugs [46]. The strongest excitatory responses were elicited by geraniol and in a lesser extent, by citronellic acid, in Dγ and Dβ sensillae, respectively [46]. In addition, studies with specific bed bug’s odorant receptors demonstrated that EOCs such as linalool, geraniol, thymol, citronellic acid elicit electrical activity on the neuron membranes housed in the olfactory sensillae [47]. The above results suggest that EOCs influence the bed bug’s nervous system inducing behavioral responses from bed bugs that reduce their exposure to EOC residues.

Due to their high volatibility, nearly all plant-derived pesticides are considered excellent fumigants with limited residual activity [18]. This assertion is not totally supported, however, by results from forced exposure bioassays that showed that residues of plant-essential oil-based products were still toxic to bed bugs after 14 d of aging [27]. Residual efficacy of essential oils is not surprising according to recent laboratory evidence that reported that some EOCs have a low evaporation rate [29,48]. If residues of EOCs maintain their toxicity over time, they could potentially be used not only to kill foraging bed bugs, but also to prevent them from reaching a host, or hiding and establishing in personal belongings. As a proof-of-concept of lasting residual effect of EOCs, we evaluated behavioral responses from both, individual and groups of bed bugs to 24-h aged residues. We selected this length of residue aging as a theoretical time to provide transient protection of personal belongings, or a sleeping host, from bed bug activity. Our behavioral data from tracking and choice tests revealed that the 1% EOCs (geraniol, eugenol, citronellic acid, and carvacrol) are avoided by bed bugs even after their residues were aged for 24 h. Interestingly, three of these EOCs, geraniol, citronellic acid and eugenol, were reported previously as having low evaporation rates during a 24 h-period (1.29%, 5.16%, and 4.48%, respectively) [29]. Avoidance response to carvacrol was expected as this component had a relative low evaporation rate (26.89%). A similar analogy can be drawn from evaluations with thymol, a highly volatile component (≥90% evaporation rate, [29], whose aged residues were not avoided in choice tests. Short repellent residual activity of some EOCs is a limiting factor for their use in urban pest control and future research should evaluate microencapsulated techniques and EOC mixtures that prolong their life action. Additionally, since the EOCs tested in this study are active ingredients of essential oils that are “generally recognized as safe” (GRAS) (https://www.fda.gov/food/generally-recognized-safe-gras/gras-substances-scogs-database, accessed on 19 February 2021), evaluation of higher concentrations of EOCs is warranted.

Contrasting behavioral responses from bed bugs were detected in evaluations with other EOCs. For example, while avoidance behavior to fresh residues of linalool was detected in individual tracking, choice tests did reveal that aged residues of this constituent induced arrestment behavior in bed bugs. Similarly, individual bed bugs visited more areas treated with aged residues of α-pinene when compared with fresh or acetone-treated areas. Although α-pinene is often included in insect baits [49], this component also exhibited strong repellency effect on house flies [50]. In mosquitoes, linalool was a strong repellent at high concentrations but had an attractive effect at low concentrations [48]. Dose-dependent behavioral responses have been reported in other arthropods [48] and these effects should receive more attention in future studies before formulating repellent products.

Although the results of avoidance responses from bed bugs to residues of EOCs are promissory for protection of personal items, they are not robust enough to be considered for general use without further context-specific testing. For example, more testing is required to determine the responses of bed bugs to EOCs in the presence of stimuli occurring in natural infestations such as bed bug aggregation pheromones, and host-associated attractants such as human odors, carbon dioxide, or heat. Information regarding host-associated attractants is crucial to determine the potential use of essential oils for personal protection against bites. In attempt to begin to elucidate these interactions, we developed an assay to determine whether barriers of EOCs would deter a bed bug from orienting to and feeding. The assay was designed following guidelines from the U.S. Environmental Protection Agency (EPA) for testing methods for bed bug repellents [51] and consists of a host-mimicking feeding assay that uses heat, a short-range attractant [52]. In our assay, bed bugs would need to cross a paper band treated with EOCs to reach the heated blood meal. We released the bugs within the first hour of the scotophase and ran the bioassay for 12 h to represent the approximate time a bed bug would forage for a host [2]. Overall, bands impregnated with fresh residues of geraniol, eugenol, carvacrol, and citronellic did not prevent hungry bed bugs from crossing and acquiring a blood meal. However, a reduction in the bed bugs’ feeding rate was observed in assays with citronellic acid, which suggest that this component might alter blood uptake. Similar inhibitory feeding effects have been observed in the kissing bug, *Triatoma rubida* (Uhler), exposed to citronella oil [25]. Regardless, our results indicated that attraction of bed bugs to a close-range heat source may take precedence over avoidance responses to EOCs, and similar responses have been also reported when essential oil-based products were tested in the presence of carbon dioxide [27]. Therefore, little benefit of protection against bed bug bites can be expected when EOC-based products are applied to items present in close proximity of a sleeping host such as mattress covers, liners, or around the bed. Further studies that evaluate responses of bed bugs to EOCs in the presence of human attractants (e.g., body odors) are required to determine the potential of EOC-based products for protecting luggage and personal items from hitchhikers.

## 5. Conclusions

Bed bugs have behavioral mechanisms that reduce their exposure to EOCs. These avoidance responses, and their implications for potential use in bed bug control programs, may vary depending on several factors including, type of EOC, age of residues, and the presence of other stimuli in the environment. Our findings suggest that novel essential oil-based formulations that include geraniol, eugenol, carvacrol, and citronellic acid have the potential to protect personal belongings. Development of potentially efficacious oil-based formulations for bed bug control might also include evaluations of higher concentrations of EOCs, and synergistic effects of mixtures of constituents.

## Figures and Tables

**Table 1:** Behavior of bed bugs recorded in arenas with fresh or 24-h aged residues of 11 essential oil constituents.

Chemical Categories	Essential Oil Component	Parameter	Control (Acetone)	Fresh Residues	24-h Aged Residues
Terpenoids	Geraniol	Distance (cm)	1.68 ± 0.08 a	2.13 ± 0.04 bA	2.09 ± 0.04 bA
		Number of visits	9.6 ± 2.03 a	1.1 ± 0.31 bAB	2.6 ± 0.73 bA
	Carvacrol	Distance (cm)	2.31 ± 0.04 a	2.73 ± 0.07 bBC	2.35 ± 0.05 aAB
		Number of visits	7.83 ± 0.98 a	2.16 ± 1.42 bABC	7.0 ± 1.43 aBC
	Thymol	Distance (cm)	2.55 ± 0.15 a	3.51 ± 0.39 bD	2.88 ± 0.25 aB
		Number of visits	10.66 ± 3.21 a	1.16 ± 0.98 bABC	2.0 ± 0.73 bA
	Citronellic Acid	Distance (cm)	2.37 ± 0.12 a	2.51 ± 0.04 aAC	2.46 ± 0.05 aAB
		Number of visits	7.33 ± 3.77 a	0.33 ± 0.21 bA	1.0 ± 0.68 bA
	Linalool	Distance (cm)	2.23 ± 0.14 a	2.42 ± 0.07 aAC	2.33 ± 0.07 aAB
		Number of visits	8.83 ± 1.62 a	2.0 ± 0.51 bBC	8.16 ± 1.95 aBC
	Menthone	Distance (cm)	2.37 ± 0.08 a	2.37 ± 0.07 aAC	2.34 ± 0.08 aAB
		Number of visits	5.83 ± 2.38 a	5.16 ± 1.51 aCD	4.66 ± 1.62 aAB
Phenylpropane	Eugenol	Distance (cm)	1.66 ± 0.07 a	2.29 ± 0.04 bAB	2.14 ± 0.07 bA
		Number of visits	9.9 ± 1.64 a	0.5 ± 0.16 bA	1.50 ± 0.76 bA
Aldehyde	trans-Cinnamaldehyde	Distance (cm)	2.56 ± 0.24 a	2.93 ± 0.21 bCD	2.33 ± 0.26 aAB
		Number of visits	12.33 ± 3.06 a	0.66 ± 042bAB	11.0 ± 2.63 aBC
Terpenes	α-pinene	Distance (cm)	2.36 ± 0.09 a	2.39 ± 0.07 aAC	2.16 ± 0.07 aA
		Number of visits	5.16 ± 1.19 a	4.0 ± 0.73 aCD	12.0 ± 2.93 bC
	β-pinene	Distance (cm)	2.32 ± 0.08 a	2.37 ± 0.01 aAC	2.4 ± 0.05 aAB
		Number of visits	3.16 ± 0.87 a	2.33 ± 0.61 aBC	4.83 ± 0.90 aB
	Limonene	Distance (cm)	2.33 ± 0.05 a	2.32 ± 0.10 aAC	2.37 ± 0.10 aAB
		Number of visits	8.33 ± 1.99 a	8.83 ± 2.72 aD	8.33 ± 3.30 aBC

Lower case letters are for mean comparison within each treatment (rows), while capital letters are for mean comparison among treatments (columns).
